# Enduring Outcomes of COVID-19 Work Absences on the US Labor Market

**DOI:** 10.1001/jamanetworkopen.2025.36635

**Published:** 2025-10-10

**Authors:** Julia M. Dennett, Evan J. Soltas, Gopi Shah Goda, Thomas A. Thornhill, Kevin Werner, Gregg S. Gonsalves

**Affiliations:** 1 Department of Epidemiology of Microbial Diseases, Public Health Modeling Unit, Yale School of Public Health, New Haven, Connecticut; 2 Now with Hasso Plattner Institute, Potsdam, Germany; 3 Department of Economics, Princeton University, Princeton, New Jersey; 4 School of Public and International Affairs, Princeton University, Princeton, New Jersey; 5 Brookings Institution, Washington, DC; 6 Urban Institute, Washington, DC

## Abstract

**Importance:**

Although the adverse outcomes of the COVID-19 pandemic on the US labor market have been well documented, the impacts of ongoing SARS-CoV-2 circulation are less clear.

**Objective:**

To determine the extent to which COVID-19 continues to generate work absences and decrease labor force participation beyond the pandemic period in the US.

**Design, Setting, and Participants:**

This cohort study used monthly data from employed survey respondents in the nationally representative Current Population Survey spanning January 2010 to December 2024 and linked to COVID-19 wastewater surveillance data from the Centers for Disease Control and Prevention. The setting was the US before the COVID-19 pandemic (before March 2020), during the pandemic (March 2020 to April 2023), and after the end of the public health emergency declaration (May 2023 to December 2024).

**Exposures:**

COVID-19 prevalence as measured by wastewater viral activity levels and comparisons across the periods before, during, and after the pandemic.

**Main Outcomes and Measures:**

The primary outcomes were health-related absences from work and subsequent labor force exits. The association between health-related absences and all measures of wastewater viral activity was analyzed using state-level data and linear regressions with state and month fixed effects.

**Results:**

The study cohort was the employed US population and represented approximately 158.4 million workers in February 2020. At this baseline, approximately 35% of workers (55.3 million workers) were aged 15 to 34 years, 41% (65.1 million workers) were aged 35 to 54 years, and 24% (38.0 million workers) were aged 55 years and older; 48% (75.2 million workers) were female; 1% (1.7 million workers) were American Indian, 7% (10.5 million workers) were Asian, 18% (27.7 million workers) were Hispanic, 11% (18.1 million workers) were non-Hispanic Black, and 62% (97.9 million workers) were non-Hispanic White; and 33% (52.5 million workers) had educational attainment of high school or less. Adverse outcomes from the COVID-19 pandemic persisted, although at attenuated levels compared with the acute pandemic period. Health-related absences from work continued to track COVID-19 circulation and were 12.9% higher in the postpandemic period compared with before the pandemic (140 000 monthly absences). Workers in occupations at greater risk of exposure and some demographic groups continued to experience elevated levels of absences in the postpandemic period. Specifically, absences were 8.1% higher for workers in low work-from-home occupations and 12.5% higher for workers in high physical proximity occupations compared with prepandemic values. Labor force exits after a health-related absence also continued to be elevated, with 13.1% more exits in the postpandemic period compared with before the pandemic (13 500 monthly exits).

**Conclusions and Relevance:**

In this cohort study of employed workers in the US, the new year-round baseline for work absences appeared to be on par with the levels formerly confined to prepandemic influenza season conditions. Policymakers should consider the consequences for workers, including the value of policies and actions that mitigate the spread of COVID-19 in the workplace. This study additionally highlights the potential for using nationally representative labor market data to monitor the impacts of public health crises.

## Key Points

**Question:**

Following the COVID-19 pandemic, has SARS-CoV-2 circulation been associated with health-related absences from work and labor force exits?

**Findings:**

In this nationally representative cohort study of approximately 158.4 million workers, rates of health-related work absences remained elevated after the pandemic and were associated with circulating SARS-CoV-2 and subsequent decreases in labor force participation by absence-affected workers.

**Meaning:**

These findings suggest that COVID-19 may have created a new year-round baseline for work absences that is similar to influenza season conditions before the pandemic; policymakers should consider expanding interventions and data collection efforts to address the negative impacts of COVID-19 on the labor force.

## Teaser

This cohort study examines the extent to which COVID-19 continues to generate work absences and decrease labor force participation beyond the pandemic period in the US.

## Introduction

The COVID-19 pandemic imposed a tremendous health burden on the US. Between early 2020 and the end of the public health emergency (PHE) declaration on May 11, 2023, more than 1.1 million US individuals died from COVID-19.^1^ COVID-19 also adversely affected the labor market during this period. Previous studies using data from the first 2 years of the pandemic found large increases in work absences because of illness, childcare, or other family or personal reasons.^2,3^ Moreover, research determined that workers with week-long COVID-19–related work absences were 7 percentage points more likely than workers without such absences to exit the labor force entirely.^2^

However, the extent to which ongoing SARS-CoV-2 transmission continues to affect the US labor market is less clear. Although macroeconomic indicators of labor market conditions have recovered, approximately 87 000 additional US individuals have died from COVID-19 since the end of the PHE.^1^ Persistent disruptions related to COVID-19 may harm the productivity of the labor force and suggest a potential role for policy that provides additional benefits and protections for workers from infection with SARS-CoV-2. Furthermore, insights from any ongoing interference due to COVID-19 can inform planning and data collection to mitigate the long-term impacts of future public health crises.

In this study, we investigated the extent to which COVID-19 illnesses continue to generate work absences and to diminish labor force participation. We focused on the association of COVID-19 with week-long health-related absences from work using nationally representative labor market survey data through December 2024. This study period spans the entirety of the pandemic period and more than a year and a half after the end of the PHE, extending previous research based on data through June 2022.^2^

## Methods

### Data Sources

This cohort study was determined to be not human participants research by the Yale University institutional review board. Data were deidentified and publicly available, so informed consent was not needed, in accordance with 45 CFR §46. This study follows the Strengthening the Reporting of Observational Studies in Epidemiology (STROBE) reporting guidelines for cohort studies. We collected data on workers from the Integrated Public Use Microdata Series (IPUMS) Current Population Survey (CPS) Basic Monthly files, a large, nationally representative sample of the US resident population.^4^ These data are created by the US Census Bureau and the Bureau of Labor Statistics and are primarily used for labor market surveillance and statistics. We obtained data spanning January 2010 to December 2024 for all US states and limited our primary analysis sample to the employed population. In the event study of absences described below, we limited our analysis sample to the employed population in the given reference month. Employment and labor force participation status may vary outside of the reference month for the event study.

We used IPUMS CPS data to identify health-related absences from work. Health-related absences are determined from civilian workers aged 15 years and older who indicated that they are currently employed but worked zero hours in the CPS reference week (which is defined as the calendar week that includes the 12th day of the month) because of “own illness/injury/medical problems.”^2,5^ The IPUMS CPS also contains detailed information on occupation and demographic characteristics of survey participants. Data on race and ethnicity (categorized as American Indian, Asian, Hispanic, non-Hispanic Black, non-Hispanic White, and other [defined as a survey respondent reporting ≥2 races]) are included in this study to consider disparities in outcomes on these demographics attributes.

To represent the circulation of SARS-CoV-2, we use Centers for Disease Control and Prevention (CDC) wastewater viral activity levels. These surveillance data are available at the national level and state level beginning in January 2022 (although not all states provided data for the full period).^6,7^ Activity levels range from less than 1.5 (index value, meaning very low) to greater than 8 (very high).^8^ We collected wastewater viral activity levels that correspond to the reference week in the CPS data. In supplemental analyses, we also use alternative sources of COVID-19 data, including COVID-19 case numbers and COVID-19–associated hospitalization rates (although these are not our preferred measures because of data limitations), as well as CDC wastewater viral activity levels for influenza A and respiratory syncytial virus (RSV). See the eAppendix in Supplement 1 for additional information on all measures of viral circulation. Note that the CDC revised the methods used to generate wastewater data in August 2025.^9^

### Statistical Analysis

To examine the association between COVID-19 and ongoing labor market disruptions, we evaluated the association between health-related absences from work and COVID-19 wastewater viral activity levels. We first compared excess health-related absences in the national CPS sample to SARS-CoV-2 circulation at the national level. Excess health-related absences were calculated by subtracting monthly averages in health-related absences from before the pandemic (January 2010 to February 2020) from the actual number of health-related absences after the onset of the pandemic (March 2020 and later) using the residuals from a linear regression of the number of health-related absences on month dummies. Owing to the flat trend in health-related absences during the prepandemic period, excess health-related absences before March 2020 represent a baseline centered around zero.

We also compared state-level variation in health-related absences and COVID-19 wastewater viral activity levels. Following Cattaneo et al,^10^ we removed state and month fixed effects and presented the results as binned scatterplots with a linear fit line. Only state-months with wastewater viral activity levels were included, resulting in an unbalanced sample (see the eAppendix in Supplement 1 for additional details). The sample with matched COVID-19 wastewater viral activity covers 91.5% of the full sample. We conducted similar national-level and state-level analyses using our secondary measures of COVID-19 severity and influenza A and RSV wastewater viral activity levels. Moreover, we analyzed the association between health-related absences and all measures of wastewater viral activity using state-level data and linear regressions with state and month fixed effects.

In addition, we evaluated heterogeneity in health-related absences across occupations at greater risk of exposure to COVID-19 and across demographic characteristics. We first identified workers who are in occupations that are more amenable to working from home and workers in occupations that place them in greater physical proximity to other people using occupational risk measures from Mongey et al.^11^ We calculated mean health-related absences before the pandemic (January 2015 to February 2020), during the pandemic (March 2020 to April 2023), and after the end of the PHE (May 2023 to December 2024). Next, we investigated heterogeneity in health-related absences across demographic characteristics. We examined health-related work absences overall and by age group, sex, race and ethnicity, and educational attainment. As above, we calculated mean health-related absences before the pandemic, during the pandemic, and after the end of the PHE, as well as the changes compared with the prepandemic period.

Finally, we examined an additional aspect of labor force disruptions: exiting the labor force after a health-related absence. We identified exits as workers with a health-related absence who indicated they were no longer in the labor force in the following month. We calculated excess labor force exits at the national level using the same method as excess health-related absences (described above). Moreover, we calculated worker-level event study estimates of the associations of a health-related absence with the probability of subsequent labor force participation. The CPS follows a rotation group structure, whereby households are interviewed over a 16-month period (interviewed for 4 months, skip 8 months, and then interviewed for another 4 months).^12^ To produce these estimates, we tracked workers over time, comparing those with health-related absences with demographically similar ones without such absences following Goda et al^2^ (see the eAppendix in Supplement 1 for additional details). We investigated participation effects at 1 month and 12 months following the absence. We also calculated the point-in-time labor-force impact of COVID-19 by combining these event-study estimates with recent monthly rates of health-related absences above the prepandemic baseline (see Goda et al^2^ for details).

We did not address multiple comparisons in our secondary outcomes, and our analyses are, therefore, exploratory. We considered a 2-sided *P* < .05 to be statistically significant. All analyses were conducted using Stata statistical software version 18.5 (StataCorp).

## Results

Our study cohort was a nationally representative sample of the employed US population, which characterized approximately 158.4 million workers at baseline in February 2020 (eTable 1 in Supplement 1). Within this cohort, roughly 35% of workers (55.3 million workers) were aged 15 to 34 years, 41% (65.1 million workers) were aged 35 to 54 years, and 24% (38.0 million workers) were aged 55 years and older; 48% (75.2 million workers) were female; 1% (1.7 million workers) were American Indian, 7% (10.5 million workers) were Asian, 18% (27.7 million workers) were Hispanic, 11% (18.1 million workers) were non-Hispanic Black, and 62% (97.9 million workers) were non-Hispanic White; and 33% (52.5 million workers) had educational attainment of high school or less.

**Figure 1.**
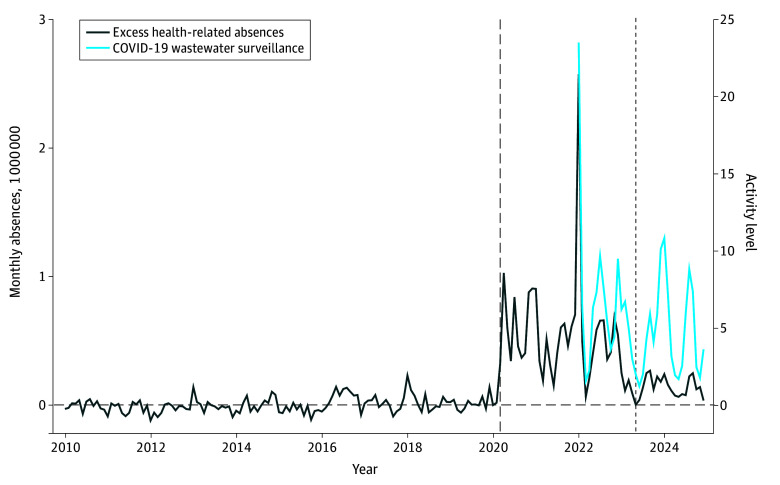
National Excess Health-Related Absences From Work vs COVID-19 Wastewater Viral Activity Levels, January 2010 to December 2024 Graph shows monthly excess health-related absences (measured in millions) for the entire US and COVID-19 wastewater viral activity levels in the reference week, which are available beginning in January 2022. Excess health-related absences were calculated by subtracting monthly averages in health-related absences from before the pandemic (January 2010 to February 2020) from the actual number of health-related absences after the onset of the pandemic (March 2020 and later). The dashed vertical line represents March 2020 (the beginning of the pandemic) and the dotted vertical line represents May 2023 (the end of the public health emergency).

We found evidence of continuing disruptions, if at a smaller scale than during the PHE. Excess health-related absences from work continued to be elevated compared with the period before the COVID-19 pandemic (Figure 1). Specifically, we observed excess health-related absences during the pandemic period (similar to previous work^2^), and also showed elevated rates that have continued since the end of the PHE. In 2024, there were, on average, approximately 1.07 million health-related absences per month. This level was comparable to peak influenza season conditions before the pandemic. For example, on average, there were 1.15 million monthly health-related absences for the December 2017 to February 2018 influenza season, 1.04 million monthly health-related absences for the December 2018 to February 2019 influenza season, and 1.06 million monthly health-related absences for the December 2019 to February 2020 influenza season.

Moreover, we found that these trends in health-related absences closely tracked COVID-19 wastewater viral activity levels since the beginning of this series in January 2022. Although excess health-related absences similarly tracked secondary COVID-19 measures (eFigure 1 in Supplement 1), they appeared less compatible with influenza A and RSV wastewater viral activity levels (eFigure 2 in Supplement 1), suggesting that COVID-19 was the reason for excess health-related absences. Compared with 1.15 million monthly absences before the pandemic, on average, there were approximately 1.79 million monthly absences during the pandemic (a 56.7% increase) and 1.29 million monthly absences after the end of the PHE (difference, 140 000 monthly absences; a 12.9% increase). These calculations also account for absences that occurred outside of the CPS reference weeks (see the eAppendix in Supplement 1 for additional details).

**Figure 2.**
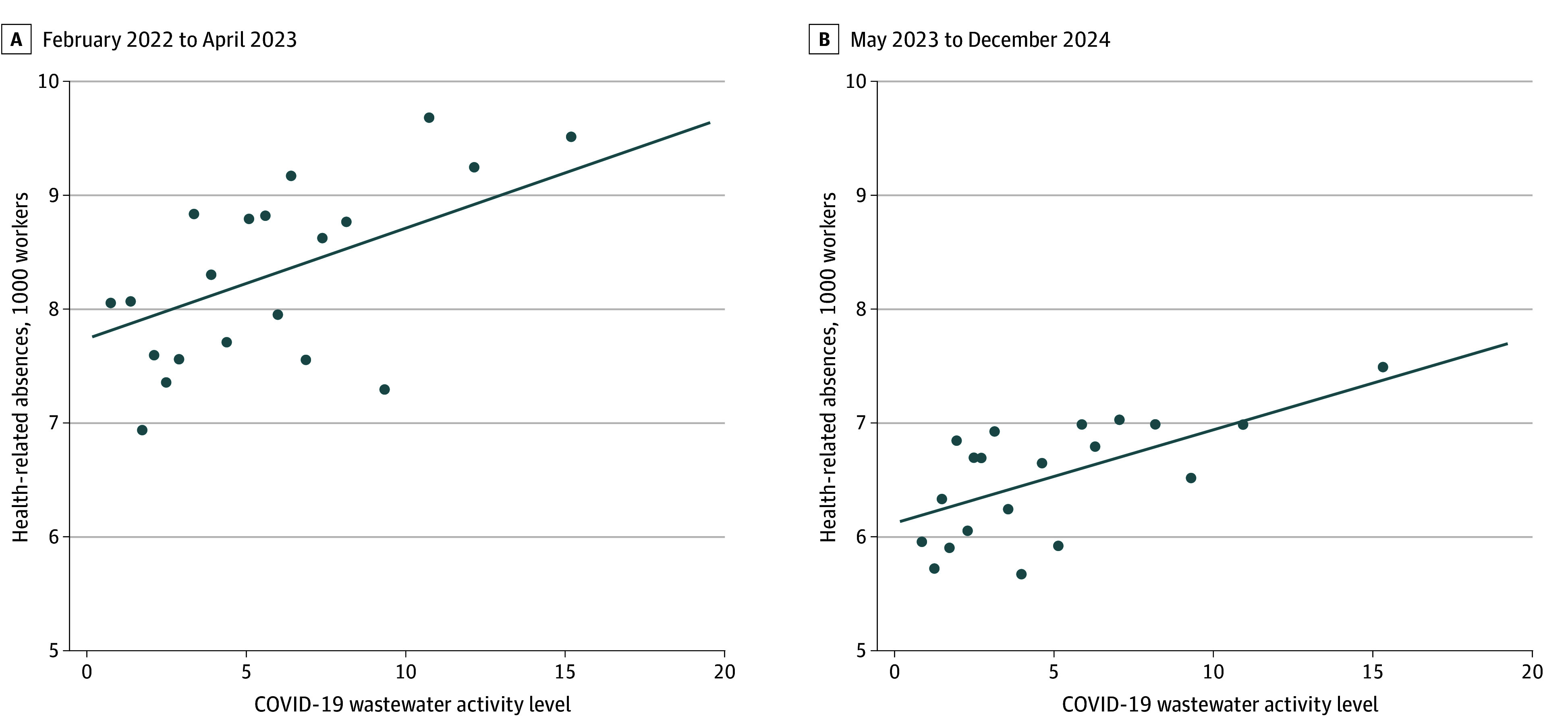
State-Level Health-Related Absences From Work vs COVID-19 Wastewater Viral Activity Levels Graphs show binned scatterplots with a linear fit of the state-level association between health-related absences vs COVID-19 wastewater viral activity levels. Displayed time periods are based on the availability of wastewater surveillance data and are divided into the period during the pandemic (February 2022 to April 2023; A) and after the end of the public health emergency (May 2023 to December 2024; B). Not all states provide data for the full period and data from North Dakota are not available.

Next, we showed that health-related absences at the state level were elevated when COVID-19 wastewater viral activity levels were also elevated, both during and after the PHE (Figure 2). As a note, we excluded wastewater data from January 2022 in this analysis; that month experienced idiosyncrasies in COVID-19 surveillance because of the introduction of the Omicron variant^13,14^ and created outliers in wastewater surveillance values (for transparency, however, we replicated this analysis including January 2022 in eFigure 3 in Supplement 1).

We saw similar results when using COVID-19 rates during the pandemic (eFigure 4 in Supplement 1, as observed in previous research^2^).We also observed a positive association between health-related absences and COVID-19 hospitalizations in 15 states during the PHE, although this association reversed itself as hospitalization rates declined after the PHE, possibly reflecting an attenuation in viral severity (eFigure 5 in Supplement 1). In contrast, we saw limited associations between health-related absences and influenza A and RSV wastewater viral activity levels (eFigures 6 and 7 in Supplement 1, respectively). In our regression analysis, we similarly observed no association between health-related absences and influenza (regression coefficient, −0.002; 95% CI, −0.014 to 0.009) or RSV (regression coefficient, 0.025; 95% CI, −0.048 to 0.097) wastewater activity, but a positive association with COVID-19 wastewater activity (regression coefficient, 0.138; 95% CI, 0.074 to 0.202) (eTable 2 in Supplement 1).

**Figure 3.**
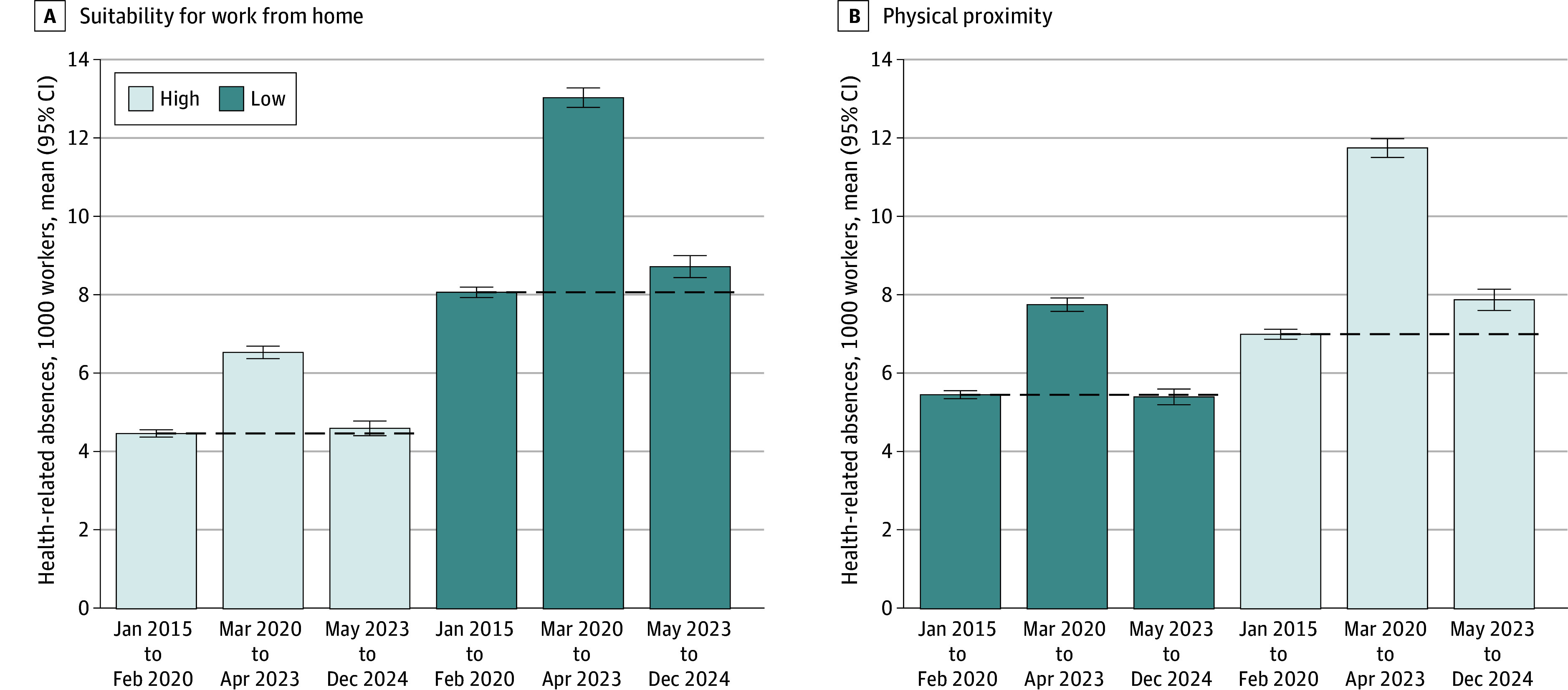
Health-Related Absences by Occupational Risk of Exposure to COVID-19 Graphs show mean health-related absences by occupational risk of exposure to COVID-19, including the occupation’s suitability for working from home (A) and physical proximity to others (B), from the period before the pandemic (January 2015 to February 2020), during the pandemic (March 2020 to April 2023), and after the end of the public health emergency (May 2023 to December 2024). Error bars indicate 95% CIs. The dashed horizontal line represents the mean value from the prepandemic period, and is included for comparison over time. Workers in low work-from-home occupations represent approximately 45.8% of the sample and workers in high physical proximity occupations represent approximately 44.5% of the sample. These classifications are missing for approximately 1.9% of the sample.

As additional evidence of continued labor force disruptions, we found that occupations with a greater risk of exposure to COVID-19 still experienced elevated health-related absences compared with before the pandemic (Figure 3). Similar to previous research,^2^ we found that workers in low work-from-home and high physical proximity occupations experienced greater increases in health-related absences during the pandemic period than workers in less risky occupations. However, we further found that this remained true after the end of the PHE; for workers in less risky occupations, health-related absences have decreased to prepandemic levels, whereas, compared with prepandemic levels, health-related absences were 8.1% higher for workers in low work-from-home occupations (8.06 absences per 1000 workers [95% CI, 7.93-8.20 absences per 1000 workers] vs 8.72 absences per 1000 workers [95% CI, 8.44-8.99 absences per 1000 workers]) and 12.5% higher for workers in high physical proximity occupations (6.99 absences per 1000 workers [95% CI, 6.87-7.12 absences per 1000 workers] vs 7.87 absences per 1000 workers [95% CI, 7.60-8.14 absences per 1000 workers]).

We also found changing patterns of health-related absence rates across demographic characteristics (Table). During the pandemic period, we found that workers who are younger, American Indian, Hispanic, or non-Hispanic Black or who have less than a bachelor’s degree experienced disproportionately large increases in health-related absences, echoing the findings of previous research.^2^ Although health-related absence rates have decreased for all demographic groups after the end of the PHE, we also saw that some groups have continued to see elevated rates compared with before the pandemic. In particular, we observed increases in workers who are younger (aged 15 to 24 years, change of 0.85 absences per 1000 workers; 95% CI, 0.34-1.37 absences per 1000 workers; aged 25 to 34 years, change of 0.70 absences per 1000 workers; 95% CI, 0.23-1.16 absences per 1000 workers), American Indian (3.36 absences per 1000 workers; 95% CI, 0.05-6.67 absences per 1000 workers), Hispanic (1.07 absences per 1000 workers; 95% CI, 0.49-1.65 absences per 1000 workers), or have some college (0.75 absences per 1000 workers; 95% CI, 0.20-1.29 absences per 1000 workers) or a bachelor’s degree (0.64 absences per 1000 workers; 95% CI, 0.21-1.07 absences per 1000 workers). However, these increases appeared to be smaller and less related to socioeconomic status compared with the pandemic-era increases.

**Table.** Health-Related Absence Rates by Demographic Characteristics^a^

Characteristic	No. of health-related absences/1000 employed workers, mean (SE)^b^			Change in rate, health-related absences/1000 employed workers, mean (SE)^b^	
	Prepandemic	Pandemic	After PHE	Prepandemic vs pandemic	Prepandemic vs after PHE
Overall	6.18 (0.06)	9.54 (0.10)	6.52 (0.12)	3.36 (0.11)^c^	0.35 (0.13)^d^
Age group, y					
15-24	3.20 (0.11)	8.57 (0.26)	4.05 (0.24)	5.37 (0.28)^c^	0.85 (0.26)^d^
25-34	3.92 (0.10)	7.15 (0.18)	4.61 (0.21)	3.23 (0.21)^c^	0.70 (0.24)^d^
35-44	5.28 (0.12)	8.07 (0.19)	4.92 (0.20)	2.79 (0.22)^c^	−0.36 (0.24)
45-54	6.72 (0.13)	9.84 (0.23)	6.96 (0.28)	3.12 (0.26)^c^	0.24 (0.31)
55-64	9.63 (0.18)	12.44 (0.28)	10.18 (0.37)	2.82 (0.33)^c^	0.55 (0.41)
≥65	12.03 (0.31)	15.71 (0.47)	12.29 (0.54)	3.69 (0.56)^c^	0.26 (0.62)
Sex					
Male	5.50 (0.08)	8.71 (0.13)	5.83 (0.15)	3.22 (0.15)^c^	0.33 (0.17)
Female	6.94 (0.09)	10.47 (0.15)	7.31 (0.17)	3.53 (0.17)^c^	0.36 (0.20)
Race and ethnicity					
American Indian	6.46 (0.56)	11.87 (1.18)	9.82 (1.59)	5.41 (1.31)^c^	3.36 (1.69)^e^
Asian	3.46 (0.17)	6.13 (0.29)	3.97 (0.34)	2.67 (0.34)^c^	0.51 (0.38)
Hispanic	5.13 (0.13)	9.84 (0.25)	6.19 (0.26)	4.71 (0.28)^c^	1.07 (0.30)^c^
Non-Hispanic Black	7.93 (0.21)	12.00 (0.36)	8.07 (0.41)	4.06 (0.42)^c^	0.13 (0.46)
Non-Hispanic White	6.38 (0.07)	9.28 (0.12)	6.50 (0.14)	2.90 (0.14)^c^	0.12 (0.16)
Other^f^	7.05 (0.53)	10.84 (0.86)	8.50 (1.12)	3.80 (1.01)^c^	1.45 (1.24)
Educational attainment					
Less than high school	7.14 (0.20)	12.39 (0.39)	7.49 (0.43)	5.25 (0.44)^c^	0.36 (0.48)
High school graduate	7.87 (0.13)	12.72 (0.23)	8.18 (0.26)	4.85 (0.26)^c^	0.31 (0.29)
Some college	7.35 (0.12)	11.84 (0.22)	8.09 (0.25)	4.50 (0.25)^c^	0.75 (0.28)^d^
College graduate	4.08 (0.10)	6.03 (0.15)	4.72 (0.19)	1.95 (0.18)^c^	0.64 (0.22)^d^
More than college	3.51 (0.12)	4.68 (0.18)	3.68 (0.21)	1.17 (0.21)^c^	0.18 (0.24)

Abbreviation: PHE, public health emergency.

^a^ The prepandemic period refers to January 2015 to February 2020, the pandemic period refers to March 2020 to April 2023, and after the end of the PHE refers to May 2023 to December 2024.

^b^ SEs are clustered by person.

^c^ *P* < .001.

^d^ *P* < .01.

^e^ *P* < .05.

^f^ Other race is defined as a survey respondent reporting 2 or more races.

**Figure 4.**
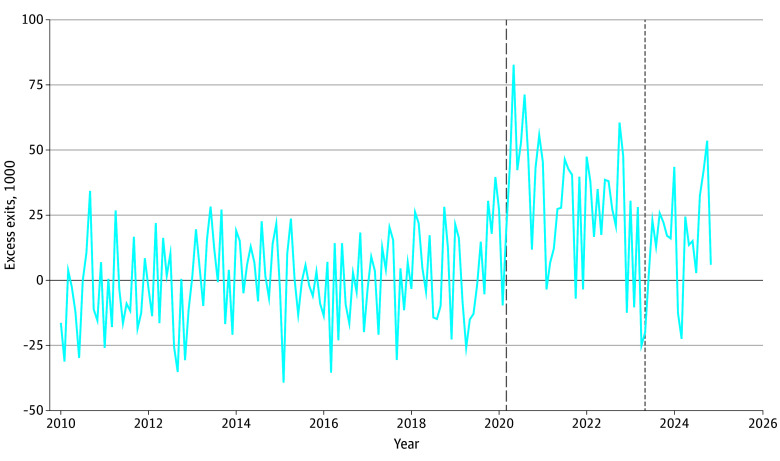
National Excess Labor Force Exits After a Health-Related Absence, January 2010 to December 2024 Graph shows monthly excess labor force exits after health-related absences (measured in thousands). These excess labor force exits were calculated by subtracting monthly averages in labor force exits from before the pandemic (January 2010 to February 2020) from the actual number of labor force exits after the onset of the pandemic (March 2020 and later). The dashed vertical line represents March 2020 (the beginning of the pandemic), and the dotted vertical line represents May 2023 (the end of the public health emergency).

Finally, we also found that health-related absences continued to have persistent impacts on labor force participation. Compared with the prepandemic baseline, labor force exits following a health-related absence were elevated during the pandemic, as previously observed,^2^ and they continued to be elevated after the end of the PHE (Figure 4). Compared with an average of approximately 103 800 monthly exits before the pandemic, there were approximately 134 200 monthly exits during the pandemic (a 29.3% increase) and 117 300 monthly exits after the end of the PHE (difference, 13 500 monthly exits; a 13.1% increase). The substantially higher exit rate rules out changes in worker propensity to take health-related absence, holding fixed the severity of underlying illness, as a plausible explanation of the increase in absences.

These aggregate patterns are consistent with event study worker-level estimates of the associations of a health-related absence with the probability of subsequent labor force participation. For cohorts of workers with absences after the PHE, we found 1-month associations that were slightly larger than those before and during the PHE (eFigure 8 in Supplement 1). Twelve-month absence associations appeared stable, although post-PHE data were limited thus far at this time horizon. Our point-in-time outcomes suggested that 49 in 10 000 US adults, or 133 000 in total, were not in the labor force in December 2024 and would have been but for COVID-19 illnesses (eFigure 9 in Supplement 1). This level of labor-force loss is approximately 19% of the peak point-in-time estimate in September 2022,^2^ and it is 41% of the estimated labor-force burden of diabetes.^15^

## Discussion

Although the COVID-19 pandemic was highly disruptive to the labor force, this cohort study found that workers continued to see elevated health-related absences linked to COVID-19 even after the end of the PHE. These were concentrated in occupations at greater risk of COVID-19 exposure. Although the pandemic-era increase in rates of health-related absence was substantial and disparate by socioeconomic status, the post-PHE increase in rates was comparatively attenuated. Health-related absences also continued to induce some workers to persistently exit the labor force, magnifying their economic costs.

Ongoing elevation in health-related absences may reflect a fundamental shift in the disease environment in the US. In particular, the introduction and continuing circulation of SARS-CoV-2 may have created a new year-round baseline for work absences that resembles prepandemic influenza season conditions. A new baseline level of work absences has important policy implications. For example, the increase in health risk has likely raised the value to workers of paid-leave policies and existing social safety net programs. Measures to reduce COVID-19 transmission in the workplace may also mitigate these new exposure risks, especially for workers in high-risk occupations. More research is needed to identify interventions that can mitigate the negative impacts of COVID-19 on the productive capacity of the labor force.

Furthermore, these results stress the need for nationally representative data collection to both understand the full implications of public health crises and inform appropriate response. In addition to the direct health consequences, the full costs include the short-term and long-term disruptions to the labor market, which may persist even after the acute phase of the crisis ends.

This study especially highlights the potential for the use of nationally representative labor market data such as the CPS to monitor the impacts of COVID-19 and other public health crises, and analyze implications for the labor force. Moreover, in future PHEs, policymakers may want monthly data on the distribution of underlying health causes of absence. Such data could be produced by adding temporary questions to the CPS for households reporting absences. Piloting such questions outside of emergency conditions would likely be feasible at low fiscal cost and would likely reduce deployment time of such questions in a future emergency.^16^

### Limitations

Use of the CPS in this context has several limitations. First, data from the CPS on health-related work absences are conditional on employment. As a result, the composition of workers represented in our sample may shift as employment rates change, particularly during the periods of high unemployment during the first year of the pandemic.^11,17^ The period after the PHE was less affected by compositional changes in the employed population, because of a rapid rebound in pandemic-exposed sectors in 2021 and 2022.^18^ Similarly, CPS data collection changed because of the pandemic, which may have altered the composition of survey participants with the largest impacts in the March 2020 to August 2020 surveys.^19^ CPS response rates have trended downward in the last decade, but decreased rapidly during the pandemic from approximately 82% in February 2020 to 65% by June before recovering to 79% by September.^20^ These disruptions were less likely to impact our post-PHE period results, although the downward trend in response rates hastened after the pandemic.^20^

## Conclusions

Ongoing SARS-CoV-2 circulation has continued to negatively affect the US labor force through 2024 by increasing health-related absences and subsequent exits from the labor market. The CPS represents an important tool to inform policy and better understand the new baseline of health risks facing US workers.

## Notes

Supplement 1. eAppendix. Supplemental methods eReferences eFigure 1. National excess health-related absences from work versus COVID-19 cases and hospitalization rate, Jan. 2010 to Dec. 2024 eFigure 2. National excess health-related absences from work versus Influenza A and RSV wastewater viral activity levels, Jan. 2010 to Dec. 2024 eFigure 3. State-level health-related absences from work versus COVID-19 wastewater viral activity levels, including Jan. 2022 eFigure 4. State-level health-related absences from work versus COVID-19 rates eFigure 5. State-level health-related absences from work versus COVID-19 associated hospitalization rates eFigure 6. State-level health-related absences from work versus Influenza A wastewater viral activity levels eFigure 7. State-level health-related absences from work versus RSV wastewater viral activity levels eFigure 8. Estimated effects of health-related work absence on labor force participation, 1-month versus 12-month follow-up, by quarter of absence eFigure 9. Estimated point-in-time effect of COVID-19 illnesses on the U.S. labor force participation rate, 2020–2024 eTable 1. Characteristics of employed U.S. population at baseline eTable 2. COVID-19 wastewater activity, not influenza A or RSV, explains state-level health-related absences from work

Supplement 2. Data Sharing Statement
