# Supplementary material for: Enduring Outcomes of COVID-19 Work Absences on the US Labor Market
Source: JAMA Netw Open. 2025 Oct 10;8(10):e2536635. doi: 10.1001/jamanetworkopen.2025.36635 (PMC12514624; doi:10.1001/jamanetworkopen.2025.36635)
Supplement: Supplement 1. — eAppendix. Supplemental methods eReferences eFigure 1. National excess health-related absences from work versus COVID-19 cases and hospitalization rate, Jan. 2010 to Dec. 2024 eFigure 2. National excess health-related absences from work versus Influenza A and RSV wastewater viral activity levels, Jan. 2010 to Dec. 2024 eFigure 3. State-level health-related absences from work versus COVID-19 wastewater viral activity levels, including Jan. 2022 eFigure 4. State-level health-related absences from work versus COVID-19 rates eFigure 5. State-level health-related absences from work versus COVID-19 associated hospitalization rates eFigure 6. State-level health-related absences from work versus Influenza A wastewater viral activity levels eFigure 7. State-level health-related absences from work versus RSV wastewater viral activity levels eFigure 8. Estimated effects of health-related work absence on labor force participation, 1-month versus 12-month follow-up, by quarter of absence eFigure 9. Estimated point-in-time effect of COVID-19 illnesses on the U.S. labor force participation rate, 2020–2024 eTable 1. Characteristics of employed U.S. population at baseline eTable 2. COVID-19 wastewater activity, not influenza A or RSV, explains state-level health-related absences from work [file jamanetwopen-e2536635-s001.pdf]

Supplemental Online Content

Dennett JM, Soltas EJ, Goda GS, Thornhill TA, Werner K, Gonsalves GS. Enduring outcomes of COVID-19 work absences on the US labor market. *JAMA Netw Open*. 2025;8(10):e2536635. doi:10.1001/jamanetworkopen.2025.36635

eAppendix. Supplemental methods
eReferences
eFigure 1. National excess health-related absences from work versus COVID-19 cases and hospitalization rate, Jan. 2010 to Dec. 2024
eFigure 2. National excess health-related absences from work versus Influenza A and RSV wastewater viral activity levels, Jan. 2010 to Dec. 2024
eFigure 3. State-level health-related absences from work versus COVID-19 wastewater viral activity levels, including Jan. 2022
eFigure 4. State-level health-related absences from work versus COVID-19 rates
eFigure 5. State-level health-related absences from work versus COVID-19 associated hospitalization rates
eFigure 6. State-level health-related absences from work versus Influenza A wastewater viral activity levels
eFigure 7. State-level health-related absences from work versus RSV wastewater viral activity levels
eFigure 8. Estimated effects of health-related work absence on labor force participation, 1-month versus 12-month follow-up, by quarter of absence
eFigure 9. Estimated point-in-time effect of COVID-19 illnesses on the U.S. labor force participation rate, 2020–2024
eTable 1. Characteristics of employed U.S. population at baseline
eTable 2. COVID-19 wastewater activity, not influenza A or RSV, explains state-level health-related absences from work

This supplemental material has been provided by the authors to give readers additional information about their work.

© 2025 Dennett JM et al. *JAMA Network Open*.

eAppendix. Supplemental methods.

1. CDC Wastewater Viral Activity Levels

Wastewater viral activity levels measure normalized values from multiple wastewater treatment sites; they represent the linear-transformed number of standard deviations above a site's baseline value, and are geographically aggregated as the median across sites.[1] All wastewater viral activity levels are linked to the corresponding reference week in the CPS data.

Wastewater viral activity levels for COVID-19 is available at the national- and state-level beginning from the week ending on Jan. 1st, 2022 (note that not all states provide data for the full period and data is not available for North Dakota, resulting in an unbalanced panel).[2,3] States included in the sample starting Jan. 2022 were: Arizona, California, Colorado, Florida, Idaho, Illinois, Massachusetts, Michigan, Missouri, Montana, Nebraska, Nevada, New York, North Carolina, Ohio, Oklahoma, Oregon, Tennessee, Utah, Virginia, Washington, West Virginia, and Wisconsin. Additional states were included in Mar. 2022 (Kentucky, Maine, South Carolina, and Texas), Apr. 2022 (Delaware, Georgia, and Minnesota), May 2022 (Connecticut, Hawaii, Indiana, Iowa, Kansas, Louisiana, New Jersey, New Mexico, Pennsylvania, Rhode Island, and Vermont), Jun. 2022 (Alaska, Arkansas, District of Columbia, Maryland, New Hampshire, South Dakota, and Wyoming), and Aug. 2022 (Alabama and Mississippi). An activity level of less than 1.5 is considered "very low" activity; between 1.5 and 3 is "low"; between 3 and 4.5 is "moderate"; between 4.5 and 8 is "high"; and greater than 8 is "very high."[1]

Wastewater viral activity levels for Influenza A is available at the national- and state-level beginning from the week ending on Sept. 18th, 2021 (note that not all states provide data for the full period and data is not available for Montana and North Dakota, resulting in an unbalanced panel).[4,5] Oregon was initially included in the sample starting Sept. 2021. Additional states were included as follows: Mar. 2022 (California, Idaho, Kentucky, and Texas), Apr. 2022 (Florida), May 2022 (Michigan), Jun. 2022 (Colorado), Jul. 2022 (Georgia), Aug. 2022 (Alabama, Illinois, Kansas, New Jersey, Pennsylvania, and Wisconsin), Sept. 2022 (Indiana, Maine, Minnesota, and North Carolina), Oct. 2022 (New Hampshire and Utah), Dec. 2022 (Iowa, Maryland, Massachusetts, Ohio, and Virginia), Feb. 2023 (Delaware), Mar. 2023 (Vermont), Apr. 2023 (Nevada and West Virginia), May 2023 (Arkansas, Hawaii, and South Dakota), Jun. 2023 (Alaska and Tennessee), Aug. 2023 (Nebraska and New York), Sept. 2023 (Washington), Oct. 2023 (Arizona), Nov. 2023 (Connecticut, District of Columbia, Mississippi, and Wyoming), Dec. 2023 (Louisiana, New Mexico, and Oklahoma), Jan. 2024 (South Carolina), Mar. 2024 (Missouri), and Jun. 2024 (Rhode Island). An activity level of less than 1.6 is considered "very low" activity; between 1.6 and 4.5 is "low"; between 4.5 and 12.2 is "moderate"; between 12.2 and 20.1 is "high"; and greater than 20.1 is "very high."[1]

Wastewater viral activity levels for RSV is available at the national- and state-level beginning from the week ending on Mar. 5th, 2022 (note that not all states provide data for the full period and data is not available for Montana and North Dakota, resulting in an unbalanced panel).[6,7] California, Idaho, Kentucky, and Texas were initially included in the sample starting Mar. 2022. Additional states were included as follows: Apr. 2022 (Florida), May 2022 (Michigan), Jun. 2022 (Colorado), Jul. 2022 (Georgia), Aug. 2022 (Alabama, Illinois, Kansas, New Jersey, Pennsylvania, and Wisconsin), Sept. 2022 (Indiana, Maine, Minnesota, North Carolina, Oklahoma, and Oregon), Oct. 2022 (New Hampshire and Utah), Dec. 2022 (Iowa, Maryland, Massachusetts, Ohio, and Virginia), Feb. 2023 (Delaware), Mar. 2023 (Vermont), Apr. 2023 (Nevada and West Virginia), May 2023 (Arkansas, Hawaii, and South Dakota), Jun. 2023 (Alaska and Tennessee), Aug. 2023 (Nebraska and New York), Sept. 2023 (Washington), Oct. 2023 (Arizona), Nov. 2023 (Connecticut, District of Columbia, Mississippi, and Wyoming), Dec. 2023 (Louisiana and New Mexico), Jan. 2024 (South Carolina), Mar. 2024 (Missouri), and Jun. 2024 (Rhode Island). An activity level of less than 4 is considered "very low" activity; between 4 and 8 is "low"; between 8 and 12 is "moderate"; between 12 and 20 is "high"; and greater than 20 "very high."[1] Note that the CDC revised the methods used to generate wastewater data on August 15, 2025 (for additional details, please see https://www.cdc.gov/nwss/data-methods.html; authors' access date 9/8/2025).

2. Additional Sources of COVID-19 data

In supplemental analyses, we examined two additional sources of COVID-19 data that contend with data limitations.

First, we calculated data on COVID cases from the New York Times COVID tracking archive cumulative state

© 2025 Dennett JM et al. *JAMA Network Open.*

files (we also analyze rates calculated as cases per 1,000 using April 1, 2020 population data from the U.S. Census Bureau).[8,9] This data only spans the beginning of the pandemic until March 23, 2023, and thus cannot be used to analyze recent COVID-19 circulation. However, it is helpful to bridge our preferred measure with the beginning of the pandemic (as wastewater surveillance data begins on Jan 1st, 2022) and to update previous work that used this outcome for a longer period of the pandemic.[10]

Second, we used COVID-NET hospitalization surveillance data from the CDC, which provides data on the rate of COVID-19 associated hospitalizations per 100,000 population.[11] This data spans the full period from March 7, 2020 to current for most states, but is only based on data from 15 states total: California, Colorado, Connecticut, Georgia, Iowa, Maryland, Michigan, Minnesota, New Mexico, New York, North Carolina (starting Oct. 2024), Ohio, Oregon, Tennessee, and Utah.

We collected COVID-19 cases and hospitalization rates that correspond to the reference week in the CPS data.

3. Event study comparing workers with and without health-related absences

Following Goda and Soltas (2024), we compared workers with and without health-related absences using a regression with fixed effects for all observed combinations of demographic-variables. These demographic strata were formed using the following variables: age (in years), sex, race/ethnicity (non-Hispanic white, non-Hispanic Black, Hispanic, Asian, American Indian, other), education (less than high school, high school graduate, some college, bachelor's degree, more than bachelor's), and the presence of a child at home. In noted specifications, we additionally and separately control for employment and labor force participation status, their hours group (full-time or part-time), and their detailed occupation group (23 categories, as defined by the U.S. Census).

The sample is limited to workers employed in the prior month, so that it is possible to record a work absence. The regression comparison includes state-by-month fixed effects to absorb regional fluctuations. We observe survey participants up to 8 months over a 16-month period, allowing for longitudinal tracking of workers before and after the absence.

4. Estimating Health-Related Absences Outside of the CPS Reference Week

We calculate the monthly average over each period to determine that there were approximately 0.94 million, 1.47 million, and 1.06 million health related absences each month before the pandemic, during the pandemic period, and after the end of the PHE, respectively, in our analysis. These health-related absences reflect a CPS survey respondent working zero hours during the CPS reference week.

However, additional absences would have occurred outside of the CPS reference weeks. The number of these absences depend on the average duration of health-related absences; for example, if the duration of each absence is exactly one week, then these values should be multiplied by four. Goda and Soltas (2023) estimate an average duration of approximately 3.3 weeks, which indicates an undercount by 22 percent. For detailed calculations, see the end of Section 4.1 in Goda and Soltas (2023).[10]

We therefore estimate an approximate additional 0.21 million, 0.32 million, and 0.23 million absences before the pandemic, during the pandemic period, and after the end of the PHE, respectively, each month as absences that occurred outside of the CPS reference week. Incorporating these additional absences results in a total of 1.15 million, 1.79 million, and 1.29 million health-related absences before the pandemic, during the pandemic period, and after the end of the PHE, respectively.

© 2025 Dennett JM et al. *JAMA Network Open*.

5. eReferences

1. Centers for Disease Control and Prevention. About Wastewater Data. National Wastewater Surveillance System (NWSS). February 7, 2025. Accessed March 4, 2025. https://www.cdc.gov/nwss/about-data.html#data-method
2. Centers for Disease Control and Prevention. Wastewater COVID-19 National and Regional Trends. National Wastewater Surveillance System (NWSS). February 27, 2025. Accessed March 3, 2025. https://www.cdc.gov/nwss/rv/COVID19-nationaltrend.html
3. Centers for Disease Control and Prevention. Wastewater COVID-19 State and Territory Trends. National Wastewater Surveillance System (NWSS). Accessed March 3, 2025. https://www.cdc.gov/nwss/rv/COVID19-statetrend.html
4. Centers for Disease Control and Prevention. Wastewater Influenza A National Trends. National Wastewater Surveillance System (NWSS). February 27, 2025. Accessed March 3, 2025. https://www.cdc.gov/nwss/rv/InfluenzaA-nationaltrend.html
5. Centers for Disease Control and Prevention. Wastewater Influenza A State and Territory Trends. National Wastewater Surveillance System (NWSS). Accessed March 3, 2025. https://www.cdc.gov/nwss/rv/InfluenzaA-statetrend.html
6. Centers for Disease Control and Prevention. Wastewater RSV National Trends. National Wastewater Surveillance System (NWSS). February 27, 2025. Accessed March 3, 2025. https://www.cdc.gov/nwss/rv/RSV-nationaltrend.html
7. Centers for Disease Control and Prevention. Wastewater RSV State and Territory Trends. National Wastewater Surveillance System (NWSS). Accessed March 3, 2025. https://www.cdc.gov/nwss/rv/RSV-statetrend.html
8. The New York Times. Coronavirus (Covid-19) Data in the United States (Archived). Published online 2021. Accessed January 21, 2025. https://github.com/nytimes/covid-19-data
9. U.S. Census Bureau. National Intercensal Population Totals: 2010-2020. Accessed January 21, 2025. https://www.census.gov/data/tables/time-series/demo/popest/intercensal-2010-2020-national.html
10. Goda GS, Soltas EJ. The impacts of Covid-19 absences on workers. *Journal of Public Economics*. 2023;222:104889. doi:10.1016/j.jpubeco.2023.104889
11. Centers for Disease Control and Prevention. COVID-NET: Coronavirus Disease 2019 (COVID-19) Hospitalization Surveillance Network. Accessed January 22, 2025. https://covid.cdc.gov/covid-data-tracker/#covidnet-hospitalization-network

© 2025 Dennett JM et al. *JAMA Network Open*.

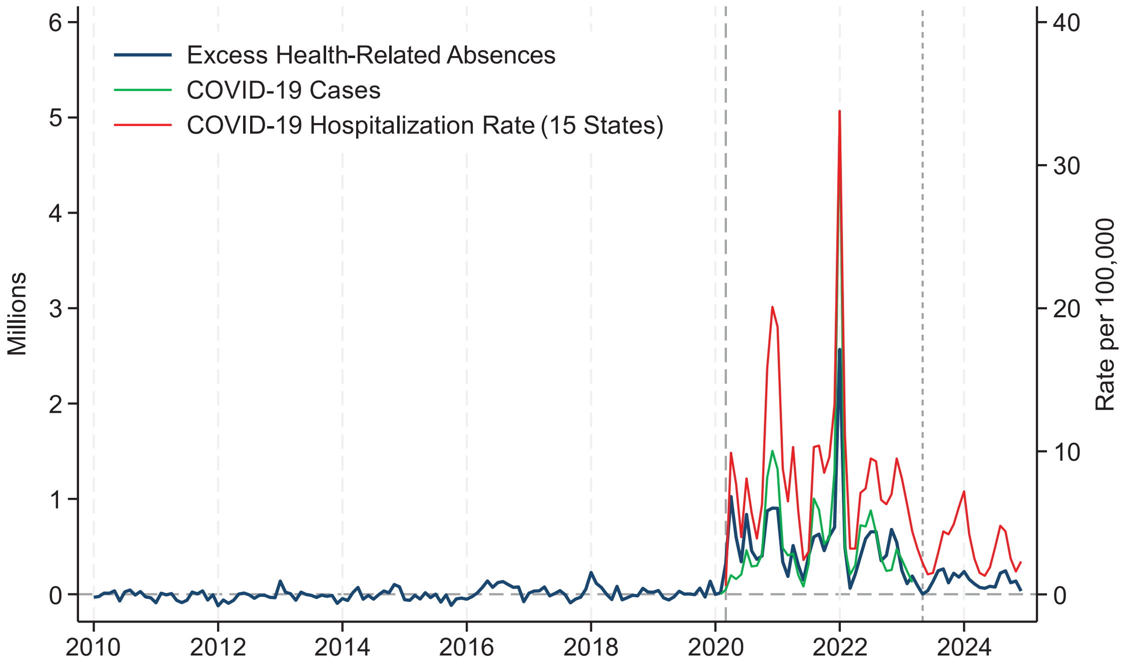

eFigure 1: **National excess health-related absences from work versus COVID-19 cases and hospitalization rate, Jan. 2010 to Dec. 2024**

Notes: This figure presents monthly excess health-related absences (measured in millions) for the entire U.S. and COVID-19 cases (measured in millions, available February 2020 to March 2023) and hospitalization rates for 15 states (measured as rate per 100,000, available March 2020 to December 2024) in the reference week. Excess health-related absences were calculated by subtracting monthly averages in health-related absences from before the pandemic (January 2010 – February 2020) from the actual number of health-related absences after the onset of the pandemic (March 2020 and later). The dashed vertical line represents March 2020 (the beginning of the pandemic) and the dotted vertical line represents May 2023 (the end of the PHE).

© 2025 Dennett JM et al. *JAMA Network Open*.

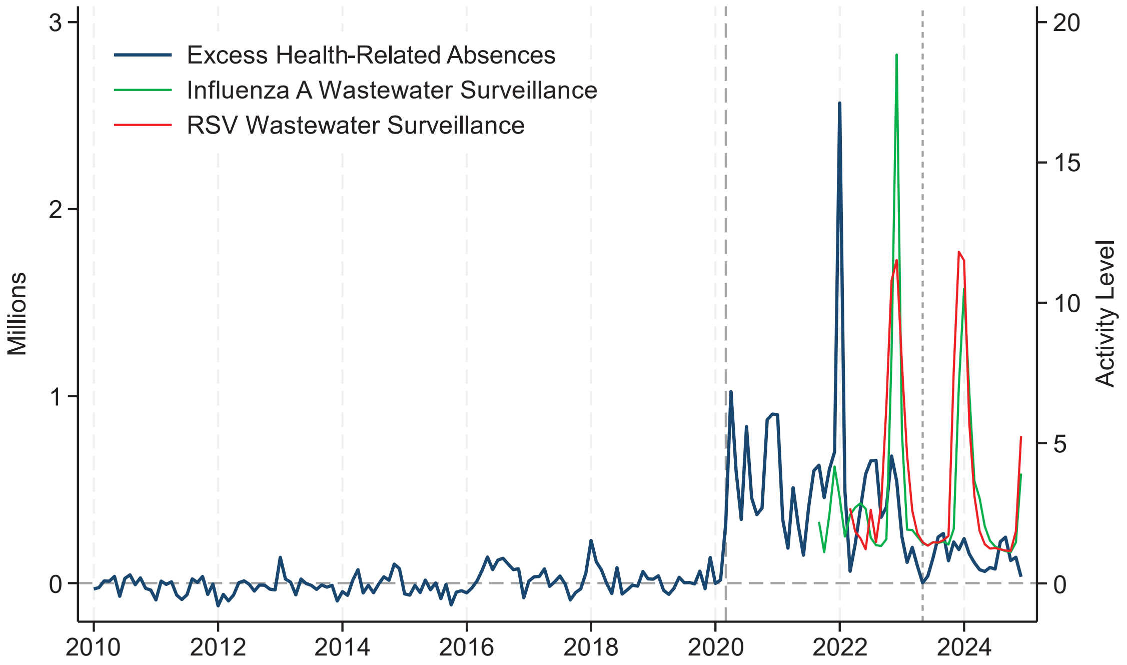

eFigure 2: National excess health-related absences from work versus Influenza A and RSV wastewater viral activity levels, Jan. 2010 to Dec. 2024

Notes: This figure presents monthly excess health-related absences (measured in millions) for the entire U.S. and Influenza A and RSV wastewater viral activity levels in the reference week, which are available beginning in Sept. 2021 and Mar. 2022, respectively. Excess health-related absences were calculated by subtracting monthly averages in health-related absences from before the pandemic (January 2010 – February 2020) from the actual number of health-related absences after the onset of the pandemic (March 2020 and later). The dashed vertical line represents March 2020 (the beginning of the pandemic) and the dotted vertical line represents May 2023 (the end of the PHE).

© 2025 Dennett JM et al. *JAMA Network Open.*

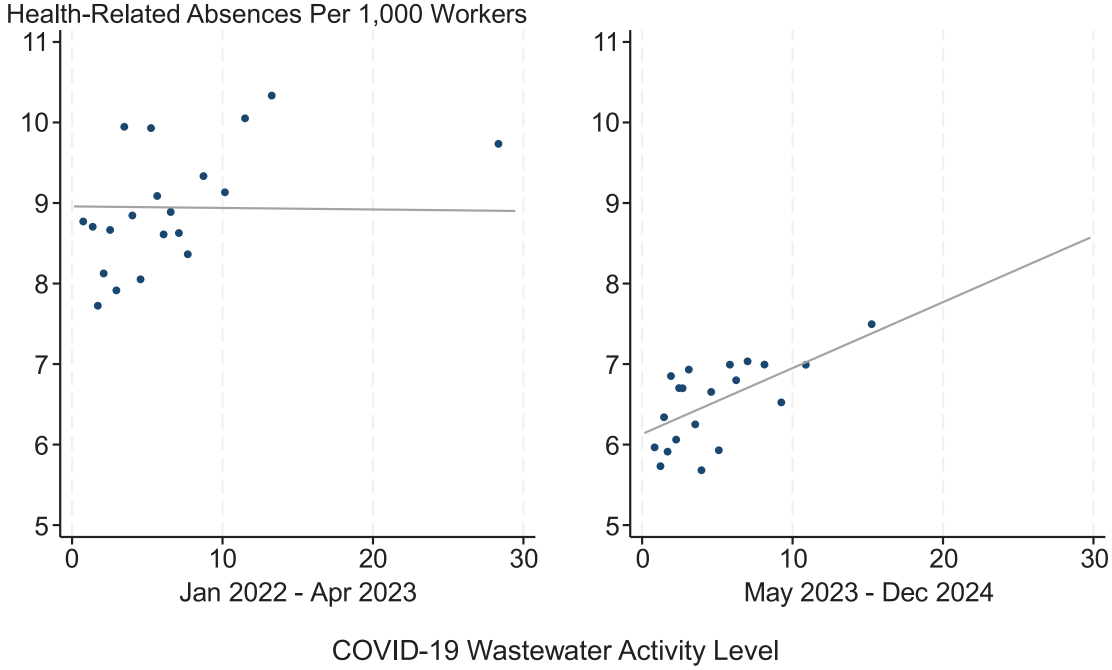

eFigure 3: State-level health-related absences from work versus COVID-19 wastewater viral activity levels, including Jan. 2022

Notes: This figure presents binned scatterplots with a linear fit of the state-level association between health-related absences versus COVID-19 wastewater viral activity levels. Displayed time periods are based on the availability of wastewater surveillance data and are divided into the period during the pandemic (January 2022-April 2023) and after the end of the PHE (May 2023-December 2024). Not all states provide data for the full period and data from North Dakota is not available.

© 2025 Dennett JM et al. *JAMA Network Open.*

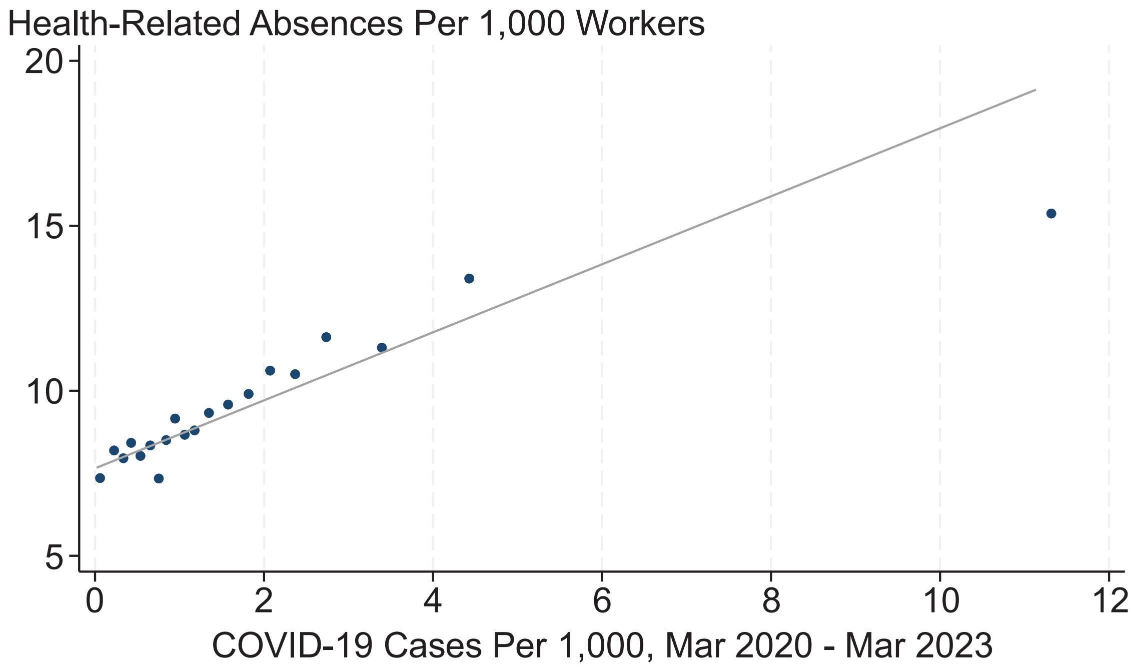

eFigure 4: State-level health-related absences from work versus COVID-19 rates

Notes: This figure presents binned scatterplots with a linear fit of the state-level association between health-related absences versus COVID-19 rates per 1,000 population. COVID-19 case data is available until March 2023 (no data is available after the end of the PHE).

© 2025 Dennett JM et al. *JAMA Network Open*.

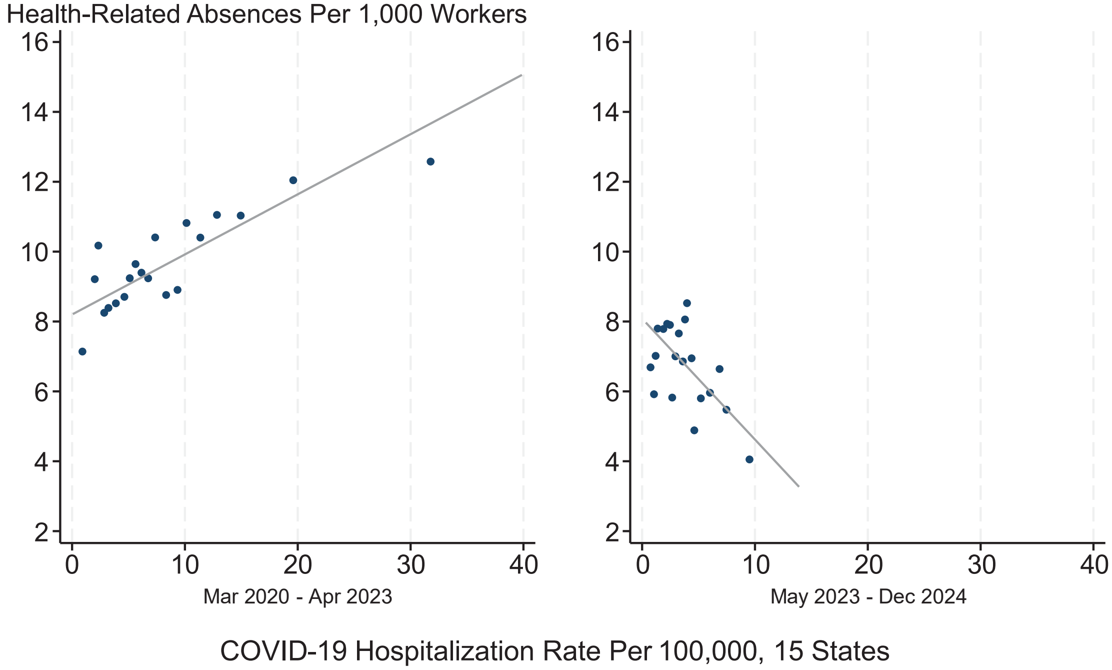

eFigure 5: State-level health-related absences from work versus COVID-19 associated hospitalization rates

Notes: This figure presents binned scatterplots with a linear fit of the state-level association between health-related absences versus COVID-19 associated hospitalization rates per 100,000 population. Displayed time periods are divided into the period during the pandemic (March 2020-April 2023) and after the end of the PHE (May 2023-December 2024). This data represents 15 states and most states provide data for the full time period.

© 2025 Dennett JM et al. *JAMA Network Open.*

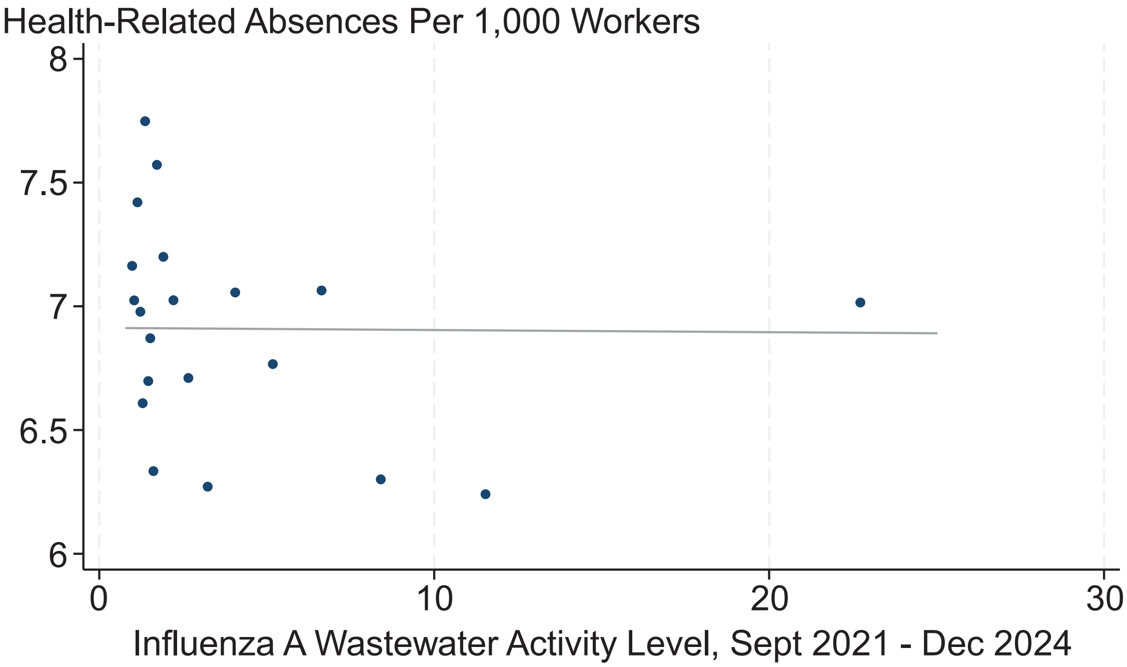

eFigure 6: State-level health-related absences from work versus Influenza A wastewater viral activity levels

Notes: This figure presents binned scatterplots with a linear fit of the state-level association between health-related absences versus Influenza A wastewater viral activity levels. Not all states provide data for the full period and data from Montana and North Dakota is not available.

© 2025 Dennett JM et al. *JAMA Network Open*.

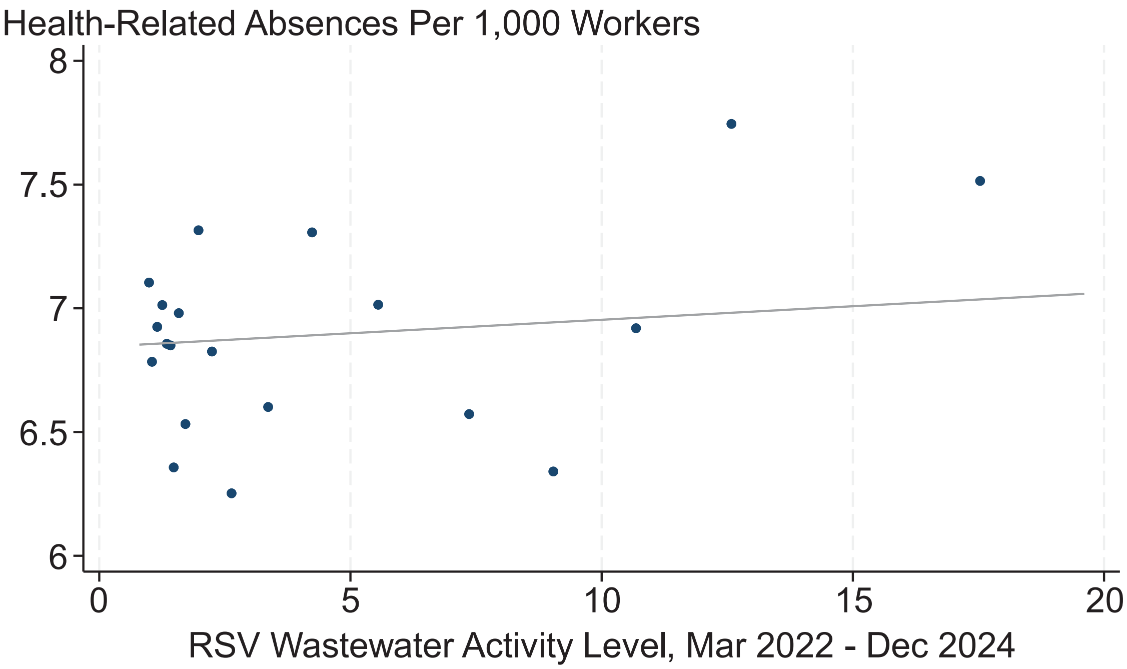

eFigure 7: State-level health-related absences from work versus RSV wastewater viral activity levels

Notes: This figure presents binned scatterplots with a linear fit of the state-level association between health-related absences versus RSV wastewater viral activity levels. Not all states provide data for the full period and data from Montana and North Dakota is not available.

© 2025 Dennett JM et al. *JAMA Network Open.*

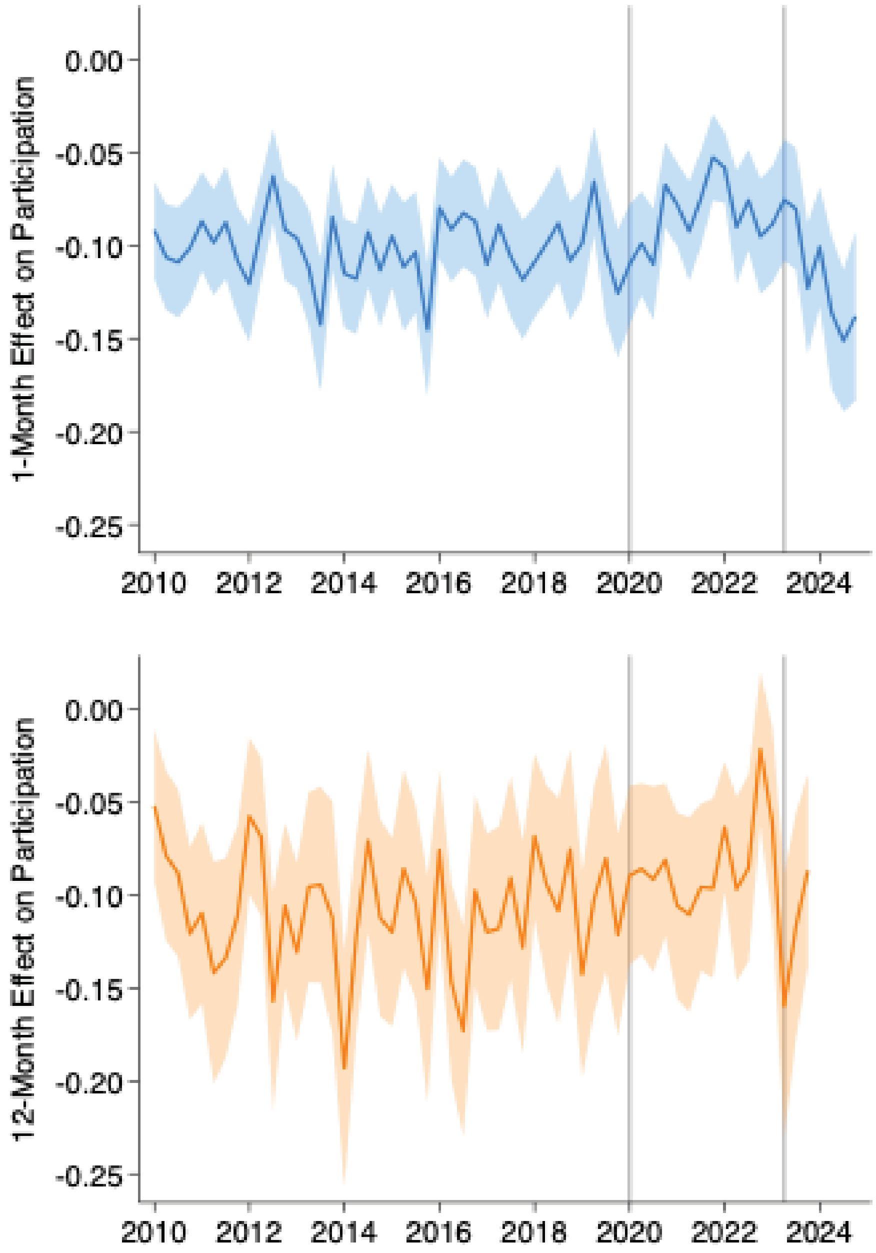

eFigure 8: Estimated effects of health-related work absence on labor force participation, 1-month versus 12-month follow-up, by quarter of absence

Notes: This table presents regression estimates of the effect of a health-related absence on the probability of labor force participation one month (top panel) and twelve months (bottom panel) after the absence. The regression specification, detailed in Goda and Soltas (2023), compares initially-employed workers with and without health-related absences who are demographically similar on observables. Bands reflect 95-percent pointwise confidence intervals, with standard errors that are clustered by person. The vertical lines represent March 2020 (the beginning of the pandemic) and the dotted vertical line represents May 2023 (the end of the PHE).

© 2025 Dennett JM et al. *JAMA Network Open*.

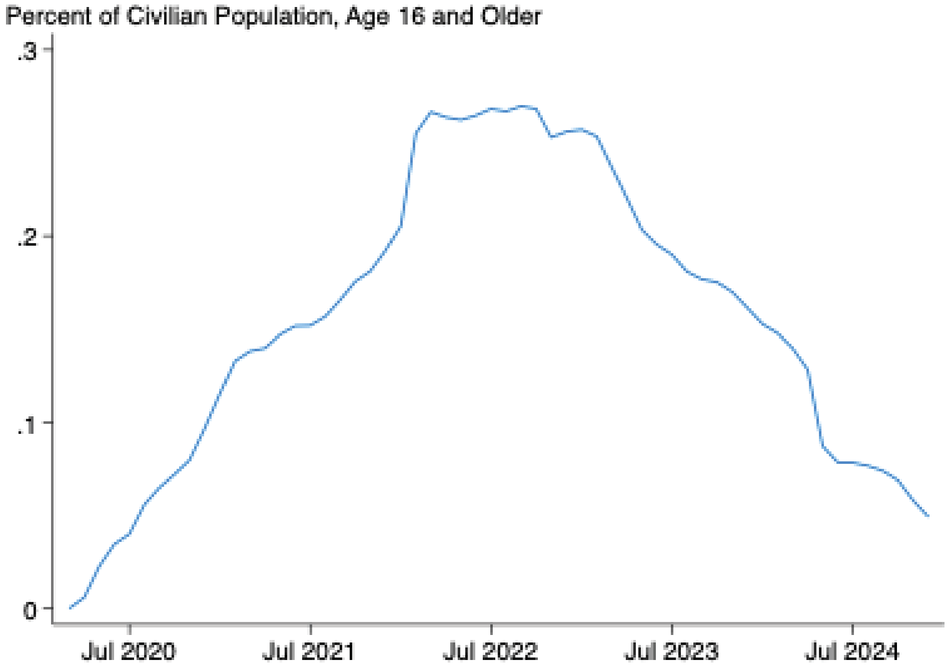

eFigure 9: Estimated point-in-time effect of COVID-19 illnesses on the U.S. labor force participation rate, 2020–2024

Notes: This table calculates the implied reduction in the U.S. labor force participation rate due to COVID-19 illnesses among initially-employed workers. To form these estimates, we combine excess health-related absences relative to the pre-pandemic baseline with event-study estimates of the participation effects of health-related absence. We then cumulate these impacts, under the assumption that the effects decay linearly after 14 months after the absence, beyond which point we cannot observe workers. For further details, see Goda and Soltas (2023).

Characteristic	Employed U.S. population February, 2020 (baseline) No. in millions (%) (N= 158.40 million)
**Age**	
15-24	19.32 (12.2)
25-34	35.93 (22.7)
35-44	33.17 (20.9)
45-54	31.95 (20.2)
55-64	27.11 (17.1)
65 plus	10.93 (6.9)
**Sex**	
Male	83.21 (52.5)
Female	75.19 (47.5)
**Race and Ethnicity**	
American Indian	1.71 (1.1)
Asian	10.51 (6.6)
Hispanic	27.65 (17.5)
Non-Hispanic Black	18.12 (11.4)
Non-Hispanic White	97.88 (61.8)
Other	2.54 (1.6)
**Educational Attainment**	
Less than High School	12.04 (7.6)
High School Graduate	40.44 (25.5)
Some College	43.22 (27.3)
College Graduate	39.80 (25.1)
More than College	22.90 (14.5)
**Health-Related Absences**	
Per Thousand Workers, Mean [95% CI]	6.69 [6.02,7.36]
**Labor Force Exit After A Health-Related Absence**	
Per Thousand Workers, Mean [95% CI]	1.04 [0.71,1.37]

eTable 1: Characteristics of employed U.S. population at baseline

Notes: This table presents summary characteristics of the employed U.S. population immediately prior to the COVID-19 pandemic (February, 2020). All variables are presented as No. in millions (%) unless otherwise indicated as Mean [95% CI]. Other race is defined as a survey respondent reporting two or more races.

© 2025 Dennett JM et al. *JAMA Network Open*.

	Dep. Var.: Health-Related Absences Per 1,000 Workers			
	(1)	(2)	(3)	(4)
**COVID-19 Wastewater**	0.136***			0.138***
	(0.032)			(0.032)
**Influenza A Wastewater**		-0.001		-0.002
		(0.006)		(0.006)
**RSV Wastewater**			0.021	0.025
			(0.036)	(0.036)
**State Fixed Effects**	Y	Y	Y	Y
**Month Fixed Effects**	Y	Y	Y	Y
**N**	1,120	1,120	1,120	1,120

eTable 2: COVID-19 wastewater activity, not influenza A or RSV, explains state-level health-related absences from work

Notes: This table presents results from regressions of the share of employed workers (per 1,000) who reported a week-long health-related absence from work in a given state and month on three wastewater viral activity measures. All regressions include state and month fixed effects and cover the common state–month sample for which all three wastewater variables are available (time period spans Mar. 2022 to Dec. 2024). Standard errors are in parentheses and are clustered by state. * p<.05, ** p<.01, *** p<.001.

© 2025 Dennett JM et al. *JAMA Network Open.*
