# Supplementary material for: Enduring Outcomes of COVID-19 Work Absences on the US Labor Market
Source: JAMA Netw Open. 2025 Oct 10;8(10):e2536635. doi: 10.1001/jamanetworkopen.2025.36635 (PMC12514624; doi:10.1001/jamanetworkopen.2025.36635)
Supplement: Supplement 2. — Data Sharing Statement [file jamanetwopen-e2536635-s002.pdf]

Data Sharing Statement

Dennett. Enduring Outcomes of COVID-19 Work Absences on the US Labor Market. *JAMA Netw Open*. Published October 10, 2025. doi:10.1001/jamanetworkopen.2025.36635

Data
Data available: Yes
Data types: Data (not involving human participants), Other (please specify)
Additional Information: All data and code will be made publicly available at GitHub upon acceptance in a journal.
How to access data: https://github.com/gregggonsalves/COVIDHRA
When available: With publication

Supporting Documents
Document types: Statistical/analytic code
How to access documents: https://github.com/gregggonsalves/COVIDHRA
When available: With publication

Additional Information
Who can access the data: Anyone requesting the data.
Types of analyses: Any purpose.
Mechanisms of data availability: https://github.com/gregggonsalves/COVIDHRA
